# Dose‐dependent efficacy and safety of centanafadine in patients with attention‐deficit/hyperactivity disorder: A systematic review of the latest evidence

**DOI:** 10.1002/pcn5.70428

**Published:** 2026-10-14

**Authors:** Daud Gul, Sonia Khan, Kushal Baral, Ziad Saber, Arindam Ghosh, Eemaz Nathaniel, Onuwabhagbe Daniel Omomhenle, Nathaniel Oluwakayode Oyedeji, Lana Omar Saed Alkhatib, Lina Jamal Amin Sa'doun, Austin Nwawueze, Muhammad Naveed Ullah Khan, Raouf Khan

**Affiliations:** ^1^ Department of Medicine Bridgeport Medical Clinic Waterloo Ontario Canada; ^2^ Department of Medicine Ibn‐e‐Sina University Mirpurkhas Pakistan; ^3^ Department of Medicine Beni Provincial Hospital Dhaulagiri Nepal; ^4^ Department of Medicine Xavier University School of Medicine Oranjestad Aruba; ^5^ Department of Medicine Dr. B.C. Roy Multi‐Speciality Medical Research Centre, IIT Kharagpur India; ^6^ Department of General Medicine/Cardiology University Hospital Wishaw Scotland UK; ^7^ Department of Psychiatry Tees, Esk and Wear Valleys NHS Foundation Trust Middlesbrough UK; ^8^ Department of Medicine Yarmouk University Irbid Jordan; ^9^ Department of Old Age Psychiatry Cumbria, Northumberland, Tyne and Wear NHS Foundation Trust Newcastle Upon Tyne UK; ^10^ Department of Forensic Psychiatry South West London and St George's Mental Health NHS Trust London UK; ^11^ Department of Medicine Comilla Medical College Comilla Bangladesh

**Keywords:** ADHD, ADHD‐RS‐5, AISRS, attention‐deficit/hyperactivity disorder, centanafadine, dose‐dependent efficacy, nonstimulant, risk of bias, safety, systematic review, tolerability

## Abstract

Centanafadine is a multimodal norepinephrine–dopamine–serotonin reuptake inhibitor (NDSRI) that has been evaluated for the treatment of attention‐deficit/hyperactivity disorder (ADHD) across pediatric, adolescent, and adult populations. This systematic review aimed to evaluate the efficacy, safety, and tolerability of centanafadine compared with placebo, with particular emphasis on dose–response patterns across different age groups. PubMed/MEDLINE, Embase, Scopus, and the Cochrane Central Register of Controlled Trials (CENTRAL) were searched from database inception to July 2026. Randomized, double‐blind, placebo‐controlled trials evaluating centanafadine in patients with ADHD were eligible for inclusion. The Cochrane Risk of Bias 2 (RoB 2) tool was used to assess the risk of bias. Because of differences in populations, formulations, dosing regimens, outcome measures, and reported effect estimates, findings were synthesized narratively rather than quantitatively. Four randomized trials reported in three publications, including 1845 randomized participants, were included. In children, high‐dose centanafadine significantly improved attention‐deficit/hyperactivity disorder rating scale, version 5 (ADHD‐RS‐5) scores compared with placebo (−16.3 vs. −10.8; *p* < 0.001), whereas the low‐dose group did not demonstrate a statistically significant difference (mean difference [MD] −2.7; 95% confidence interval [CI], −6.0 to 0.5; *p* = 0.10). In adolescents, 328.8 mg significantly improved ADHD‐RS‐5 scores compared with placebo (−18.5 vs. −14.2; *p* = 0.0006; Cohen's *d* = −0.40), whereas 164.4 mg did not (*p* = 0.3016). In adults, both 200 and 400 mg/day significantly improved Adult ADHD Investigator Symptom Rating Scale (AISRS) scores, with LSMDs ranging from −2.74 to −4.42 (*p* ≤ 0.039). Treatment‐emergent adverse events occurred in 25%–39% of children, 23.8%–50.3% of adolescents, and 17.6%–31.8% of adults, with higher rates generally observed at higher doses in some populations. Centanafadine demonstrated improvement in core ADHD symptoms across pediatric, adolescent, and adult populations, with a more apparent dose–response pattern in children and adolescents and significant efficacy across the adult doses evaluated. Higher doses were associated with increased treatment‐emergent adverse events in some populations. However, the evidence base remains limited to four randomized trials from three publications, with predominantly short‐term follow‐up, no active‐comparator trials, and limited independently sponsored evidence. Therefore, the efficacy, safety, and dose–response findings should be interpreted cautiously. Longer term, independently sponsored, and head‐to‐head comparative trials are needed to further establish the optimal dosing, comparative effectiveness, and long‐term safety of centanafadine.

## INTRODUCTION

A common neurodevelopmental disorder, attention‐deficit/hyperactivity disorder (ADHD) is characterized by persistent, developmentally inappropriate patterns of impulsivity, hyperactivity, and/or inattention that impair academic, social, occupational, or everyday functioning to a clinically significant degree. Despite the fact that ADHD is often diagnosed in childhood, symptoms can last throughout adolescence and age and cause significant functional and psychological stress throughout life.^1^, ^2^

ADHD is a significant global health burden. The prevalence of ADHD in children and adolescents is estimated to be around 7.2% worldwide, according to a systematic review and meta‐analysis; estimates for adults are still quite high.^2^, ^3^ According to a global comprehensive study, the prevalence of symptomatic adult ADHD was 6.76% and persistent adult ADHD was 2.58%, underscoring the ongoing impact of ADHD after childhood.^3^ Pharmacological treatment is still a crucial part of managing ADHD. Although stimulants, especially methylphenidate and amphetamine formulations, are usually thought to be quite effective, their usage may be restricted by side effects, insufficient response, contraindications, worries about misuse or diversion, and individual tolerability.^4^, ^5^ For individuals who do not respond well to or are unable to tolerate stimulant therapy, nonstimulant drugs such as atomoxetine, guanfacine, and clonidine offer significant alternatives.^5^, ^6^

Centanafadine is a multimodal norepinephrine–dopamine–serotonin reuptake inhibitor (NDSRI) that affects three monoaminergic pathways by inhibiting the norepinephrine, dopamine, and serotonin transporters, thereby modulating attention, arousal, and behavioral control. It has been studied in children, adolescents, and adults using sustained‐release (SR) and extended‐release (ER) formulations across various doses. Differences in dose, formulation, and developmental stage are important when evaluating its efficacy and tolerability, as treatment response and adverse‐event profiles may vary according to age and drug exposure. Therefore, evaluating the dose–response relationship across different age groups is important for determining whether higher doses provide greater symptomatic improvement and whether increased efficacy is accompanied by a corresponding increase in adverse events.^7^, ^8^, ^9^

Despite the growing clinical evidence, the dose‐dependent efficacy, safety, and tolerability of centanafadine across different age groups have not been comprehensively synthesized. Therefore, this systematic review aimed to evaluate the dose‐dependent efficacy, safety, and tolerability of centanafadine in patients with ADHD, with particular emphasis on differences in treatment response across pediatric, adolescent, and adult populations and across the dosing regimens evaluated in clinical trials.

## METHODOLOGY

### Protocol and registration

This systematic review was conducted in accordance with the Preferred Reporting Items for Systematic Reviews and Meta‐Analyses (PRISMA) 2020 statement.^10^ The review protocol was prospectively registered with PROSPERO (CRD420261475007) before study selection and data extraction.

### Search strategy

A systematic literature search was performed in PubMed/MEDLINE, Embase, Scopus, and the Cochrane Central Register of Controlled Trials (CENTRAL) from database inception to July 2026. The search strategy combined centanafadine‐related terms with ADHD‐related terms using the Boolean operator AND. The centanafadine search block included “centanafadine,” “centanafadine hydrochloride,” “centanafadine XR,” “EB‐1020,” and “DOV 216,419,” while the ADHD search block included “ADHD,” “attention deficit hyperactivity disorder,” “attention‐deficit hyperactivity disorder,” and “attention deficit disorder with hyperactivity.” Database‐specific controlled vocabulary, free‐text terms, and syntax were adapted according to the indexing requirements of each database. The complete database‐specific search strategies, including the Boolean operators and search syntax, are provided in the Supplementary Material to ensure reproducibility. No restrictions were applied to publication year or outcomes.

### Eligibility criteria

The eligibility criteria have been summarized in Table 1.

**Table 1:** Aggregated Population, Intervention, Comparator, Outcomes, and Study design (PICOS) framework and eligibility criteria.

PICOS component	Eligibility criteria
Population (P)	Individuals diagnosed with ADHD across the lifespan, including children aged 6–12 years, adolescents aged 13–17 years, and adults aged 18–55 years.
Intervention (I)	Centanafadine, administered as either an extended‐release once‐daily formulation or sustained‐release twice‐daily formulation, at any reported therapeutic dose.
Comparator (C)	Placebo for randomized controlled trials or baseline values for eligible open‐label, single‐arm evaluations.
Primary efficacy outcomes (O)	Mean change from baseline in ADHD symptoms measured using validated investigator/clinician‐rated scales, specifically ADHD‐RS‐5 symptoms total raw score or AISRS total score.
Secondary efficacy outcomes (O)	CGI‐S and executive‐functioning/behavioral measures.
Safety and tolerability outcomes (O)	TEAEs, severity of adverse events, vital signs.
Study design (S)	Phase 3 clinical trials, including 6‐week randomized, double‐blind, placebo‐controlled trials and 52‐week open‐label safety‐extension studies.
Publication eligibility	Full‐text clinical trial reports with sufficient information for extraction of at least one eligible outcome.

Abbreviations: ADHD, attention‐deficit/hyperactivity disorder; ADHD‐RS‐5, attention‐deficit/hyperactivity disorder rating scale, version 5; AISRS, Adult ADHD Investigator Symptom Rating Scale; CGI‐S, Clinical Global Impression‐Severity; TEAE, treatment‐emergent adverse event.

### Inclusion criteria

A predetermined aggregated PICOS framework was used to choose the studies. The following criteria must be met for inclusion: (1) studies that enroll people with ADHD across the lifespan, including children aged 6–12, adolescents aged 13–17, and adults aged 18–55; (2) studies that evaluate centanafadine at any reported therapeutic dose, either as an ER once‐daily formulation or a SR twice‐daily formulation; (3) studies that include a placebo control or, for eligible open‐label single‐arm evaluations, comparison with baseline values; and (4) studies that report at least one prespecified efficacy, safety, or tolerability score. Treatment‐emergent adverse events (TEAEs), adverse‐event severity, and outcomes were among the safety and tolerability outcomes. Phase 3 clinical trials, such as short‐term 6‐week randomized, double‐blind, placebo‐controlled trials and long‐term 52‐week open‐label safety‐extension studies; and full‐text clinical trial reports that contained enough data to extract at least one eligible outcome.

### Exclusion criteria

The following criteria must be excluded:

(1) Studies enrolled subjects without ADHD or studies in which ADHD‐specific data could not be extracted separately; (2) studies evaluated an intervention other than centanafadine; or studies in which only a subset of subjects was treated with centanafadine; (3) studies lacking an eligible placebo or baseline comparison (except for eligible open‐label long‐term safety‐extension studies); (4) studies that did not report at least one prespecified efficacy, safety, or tolerability outcome (except for companion publications reporting additional eligible efficacy or safety data); (5) studies of children enrolled outside of the specified age range, unless the eligible age range was separately reported; (6) conference abstracts or other publications without sufficient extractable data (except for companion publications reporting additional eligible efficacy or safety information); and (7) studies for which the full text was not obtained following reasonable efforts.

### Study screening and data extraction

All the collected studies were entered into Rayyan for primary screening. Duplicate records were first removed, and then the titles and abstracts were screened independently by two reviewers for eligibility, based on predesignated eligibility criteria. These potentially relevant records were then evaluated by the same two reviewers using full‐text screening. Zotero was used as a secondary resource for reference management and screening purposes, to organize and double check bibliographic information. Disagreements between the two reviewers in the screening process were settled by discussion and consensus; if no consensus was reached, a third reviewer adjudicated the disagreements.

Two reviewers extracted the data, independently, from a standardized Microsoft Excel spreadsheet. Information extracted comprised study characteristics (author, publication year, NCT registration number, study design, comparator and follow‐up period), baseline characteristics and patient demographics (sample size, age, sex, weight, body mass index [BMI], race/ethnicity, and baseline ADHD severity measures), efficacy and safety outcomes. Data on efficacy included ADHD symptom scores, Clinical Global Impression‐Severity (CGI‐S), Conners measures, Adult ADHD Investigator Symptom Rating Scale (AISRS), and other prespecified clinical parameters; data on safety included TEAEs and severity of adverse events. Data were collected independently of each arm of the treatment and, if possible, independently of each separately reported trial. The two reviewers extracted data at the same time, and discrepancies in data extraction were compared and clarified by consensus, using a third reviewer to resolve any remaining discrepancies.

### Risk of bias assessment

Cochrane Risk of Bias 2 (RoB 2)^11^ was used to evaluate the methodological quality of the included RCTs. Five domains were assessed: (1) domain of randomization source bias, (2) domain of deviation from intended interventions source bias, (3) domain of missing outcome data source bias, (4) domain of outcome measurement source bias, and (5) domain of selection of the reported result source bias. Low risk of bias, some concerns, and high risk of bias were assigned to each of the domains, with an overall risk‐of‐bias judgment. Two Ward trials were considered to have low overall risk of bias, while both Adler trials were considered to have some concerns, mostly due to missing outcome data.

### Data synthesis

However, no quantitative meta‐analysis was conducted due to the heterogeneity of age groups included, doses of centanafadine, the measured outcomes, treatment duration, and the reporting formats. Rather, the results were qualitatively integrated. The efficacy and safety results are summarized separately for each trial and treatment dose and compared directly to placebo using effect estimates and statistical measures offered in the original publications: mean differences, least‐squares mean differences, 95% confidence intervals, *p* values, and standardized effect sizes, as available. Reported numbers and percentages of events were summarized for safety outcomes. A particular focus was to detect dose–response trends in efficacy and adverse events.

## RESULTS

### Study selection process

Four electronic databases (PubMed, Embase, Scopus, and Cochrane) were searched, yielding a total of 173 records. Following the removal of 56 duplicate records, 118 records were left for screening for title and abstract. Of these, 92 were removed, and 26 reports were available for full‐text retrieval. 26 reports were retrieved and assessed for eligibility without any failures. After full‐text assessment, 23 reports were excluded, 13 of which were for the wrong population, 7 for the wrong intervention, and 3 for the wrong outcomes. In the end, 3 studies were found to be eligible and included in the systematic review (Figure 1).

**Figure 1:**
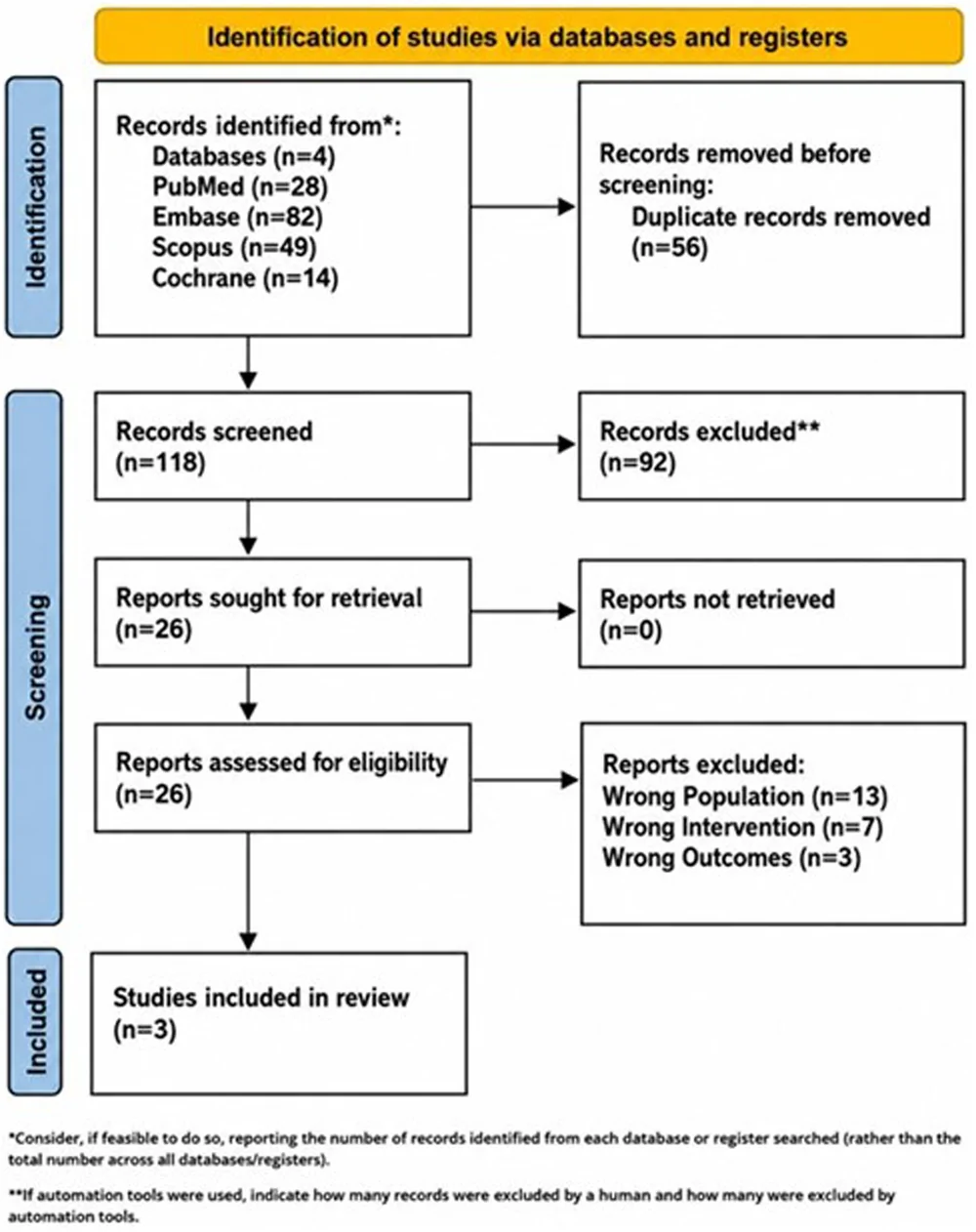
Preferred Reporting Items for Systematic Reviews and Meta‐Analyses (PRISMA). The flow diagram summarizes the identification, screening, eligibility assessment, and final inclusion of studies in the systematic review.

### Baseline characteristics and patient demographics

In the four randomized trials (three studies),^12^, ^13^, ^14^ the number of participants per treatment arm ranged from 146 to 164 children, adolescents, and adults with ADHD. The mean age was 9.1‐9.2 years in Ward et al. (2025),^12^ and 54%–65% of the participants were males; mean BMI was 19.8–20.4 kg/m^2^. In Ward et al. (2026), participants had a mean age of approximately 14.5–14.7 years, with males comprising 55.5%–63.9%, and mean BMI ranging from 23.7 to 24.3 kg/m^2^. Baseline ADHD severity scores were similar in the Ward et al. (2025) trial,^12^ with attention‐deficit/hyperactivity disorder rating scale, version 5 (ADHD‐RS‐5) scores ranging from 42.9 to 43.2 and CGI‐S‐ADHD scores ranging from 4.8 to 4.9, and were broadly similar between groups in the Conners 3‐Parent Short scores. The adults in Adler et al. (2022) Study 1^14^ had mean ages ranging from 35.0 to 36.6 years, about half were male, and baseline AISRS scores ranged from 39.4 to 39.7, with CGI‐S scores of 4.5 for all treatment groups. In Study 2 of Adler et al. (2022),^14^ age, sex, and race values were not reported for the arms and were reported as not reported (NR); mean BMI ranged from 27.1 to 28.3 kg/m^2^, and baseline AISRS scores ranged from 37.6 to 38.6, and CGI‐S scores ranged from 4.5 to 4.6 (Table 2).

**Table 2:** Study characteristics, baseline characteristics, and patient's demography.

Study	NCT	Treatment group	*N*	Age, mean (SD/range), years	Male, *n* (%)	Female, *n* (%)	Weight, mean (SD), kg	BMI, mean (SD), kg/m^2^	**Race/ethnicity, *n* (%)**	Baseline ADHD score, mean (SD)^a^	CGI‐S/CGI‐S‐ADHD, mean (SD)
Ward et al., 2025 (children)	NCT05428033	Low‐dose CTN	154	9.3 (6–12)	87 (56%)	67 (44%)	41.9 (15.4)	20.4 (4.8)	White 102 (66%); Black 40 (26%); Asian 1 (1%); AI/AN 2 (1%); NH/PI 0; Other 9 (6%)	43.1 (6.8)	4.9 (0.7)
	NCT05428033	High‐dose CTN	162	9.1 (6–12)	87 (54%)	75 (46%)	40.8 (15.2)	20.3 (4.9)	White 105 (65%); Black 47 (29%); Asian 2 (1%); AI/AN 1 (1%); NH/PI 0; Other 7 (4%)	42.9 (6.6)	4.8 (0.7)
	NCT05428033	Placebo	164	9.2 (6–12)	106 (65%)	58 (35%)	40.8 (16.0)	19.8 (4.5)	White 105 (64%); Black 44 (27%); Asian 2 (1%); AI/AN 0; NH/PI 2 (1%); Other 11 (7%)	43.2 (6.8)	4.9 (0.7)
Ward et al., 2026 (adolescents)	NCT05257265	CTN 164.4 mg	155	14.5 (1.3)	86 (55.5%)	69 (44.5%)	65.4 (17.6)	23.7 (5.5)	White 106 (68.4%); Black 43 (27.7%); Asian 4 (2.6%); Other 2 (1.3%); Hispanic 40 (25.8%)	37.7 (6.1)	4.5 (0.6)
	NCT05257265	CTN 328.8 mg	155	14.7 (1.4)	99 (63.9%)	56 (36.1%)	69.0 (19.7)	24.3 (5.6)	White 110 (71.0%); Black 36 (23.2%); AI/AN 1 (0.6%); Asian 1 (0.6%); NH/PI 1 (0.6%); Other 6 (3.9%); Hispanic 39 (25.2%)	37.8 (6.4)	4.5 (0.6)
	NCT05257265	Placebo	149	14.7 (1.3)	87 (58.4%)	62 (41.6%)	67.5 (16.9)	24.2 (5.3)	White 107 (71.8%); Black 35 (23.5%); AI/AN 3 (2.0%); Asian 1 (0.7%); Other 3 (2.0%); Hispanic 50 (33.6%)	37.0 (5.7)	4.4 (0.5)
Adler et al., 2022, Study 1 (adults)	NCT03605680	CTN 200 mg/day	154	36.6 (9.8)	78 (50.6%)	76 (49.4%)	82.6 (17.9)	28.1 (5.2)	White 126 (87.8%); Black 19 (12.3%); Asian 4 (2.6%); AI/AN 0; NH/PI 1 (0.6%); Other 4 (2.6%)	39.7 (6.7) (AISRS)	4.5 (0.6)
	NCT03605680	CTN 400 mg/day	156	35.3 (10.4)	81 (51.9%)	75 (48.1%)	81.8 (16.1)	27.8 (5.0)	White 123 (78.8%); Black 23 (14.7%); Asian 2 (1.3%); AI/AN 3 (1.9%); NH/PI 1 (0.6%); Other 4 (2.6%)	39.4 (6.8) (AISRS)	4.5 (0.6)
	NCT03605680	Placebo	156	35.0 (9.9)	80 (51.3%)	76 (48.7%)	81.4 (18.7)	27.9 (5.3)	White 130 (83.3%); Black 21 (11.5%); Asian 4 (2.6%); AI/AN 0; NH/PI 0; Other 1 (0.6%)	39.4 (7.1) (AISRS)	4.5 (0.6)
Adler et al., 2022, Study 2 (adults)	NCT03605836	CTN 200 mg/day	147	34.5 (9.7)	76 (51.7%)	71 (48.3%)	82.7 (18.2)	28.0 (5.4)	White 112 (76.2%); Black 17 (11.9%); Asian 10 (6.8%); NH/PI 1 (0.7%); Other 7 (4.8%); Hispanic 27 (18.4%)	37.6 (6.7) (AISRS)	4.6 (0.6)
	NCT03605836	CTN 400 mg/day	147	35.2 (10.4)	77 (52.4%)	70 (47.6%)	84.2 (20.4)	28.3 (5.4)	White 120 (81.6%); Black 19 (12.9%); Asian 2 (1.4%); AI/AN 2 (1.4%); Other 4 (2.7%); Hispanic 23 (15.6%)	38.6 (7.0) (AISRS)	4.6 (0.5)
	NCT03605836	Placebo	146	35.2 (9.6)	80 (54.8%)	66 (45.2%)	80.6 (17.1)	27.1 (5.0)	White 114 (78.1%); Black 21 (11.4%); Asian 6 (4.1%); AI/AN 2 (1.4%); Other 3 (2.1%); Hispanic 29 (19.9%)	37.8 (6.5) (AISRS)	4.5 (0.6)

Abbreviations: ADHD, attention‐deficit/hyperactivity disorder; AI/AN, American Indian/Alaska Native; AISRS, Adult ADHD Investigator Symptom Rating Scale; BMI, body mass index; CGI‐S, Clinical Global Impression‐Severity; CTN, centanafadine; NH/PI, Native Hawaiian/Other Pacific Islander.

^a^ For Adler Study 2, the source available here reports overall age/sex characteristics, but the treatment‐arm‐specific values were not available in the extracted material; therefore, I have deliberately used not reported (NR) rather than estimating them. The adult Study 2 source confirms the treatment‐arm sample sizes and reports the treatment‐arm weight, BMI, AISRS, subscale, ASRS, and CGI‐S values.

### Risk of bias assessment using ROB 2.0

The Cochrane RoB 2 tool was used to evaluate the risk of bias in five domains. The risk of bias was found to be low for both Ward et al. (2025)^12^ and Ward et al. (2026)^13^ in all five domains: randomization process, deviations from intended interventions, missing outcome data, outcome measurement, and selection of the reported result. Adler et al., Study 1 and Study 2,^14^ however, were rated with some concerns overall, with the specific domain of missing outcome data being the reason. The randomization, deviations from intended interventions, measurement of outcomes, and selection of the reported result were considered to be at low risk of bias in both Adler trials, with some concerns regarding missing outcome data. In Study 1, 466 participants were randomized, 348 (74.7%) of whom completed the trial; in Study 2, 440 participants were randomized, 336 (76.4%) of whom completed the trial. Overall, 50% of the included trials were considered to be low risk of bias, 50% were considered to be some concerns, and none were considered to be high risk of bias (Figures 2 and 3).

**Figure 2:**
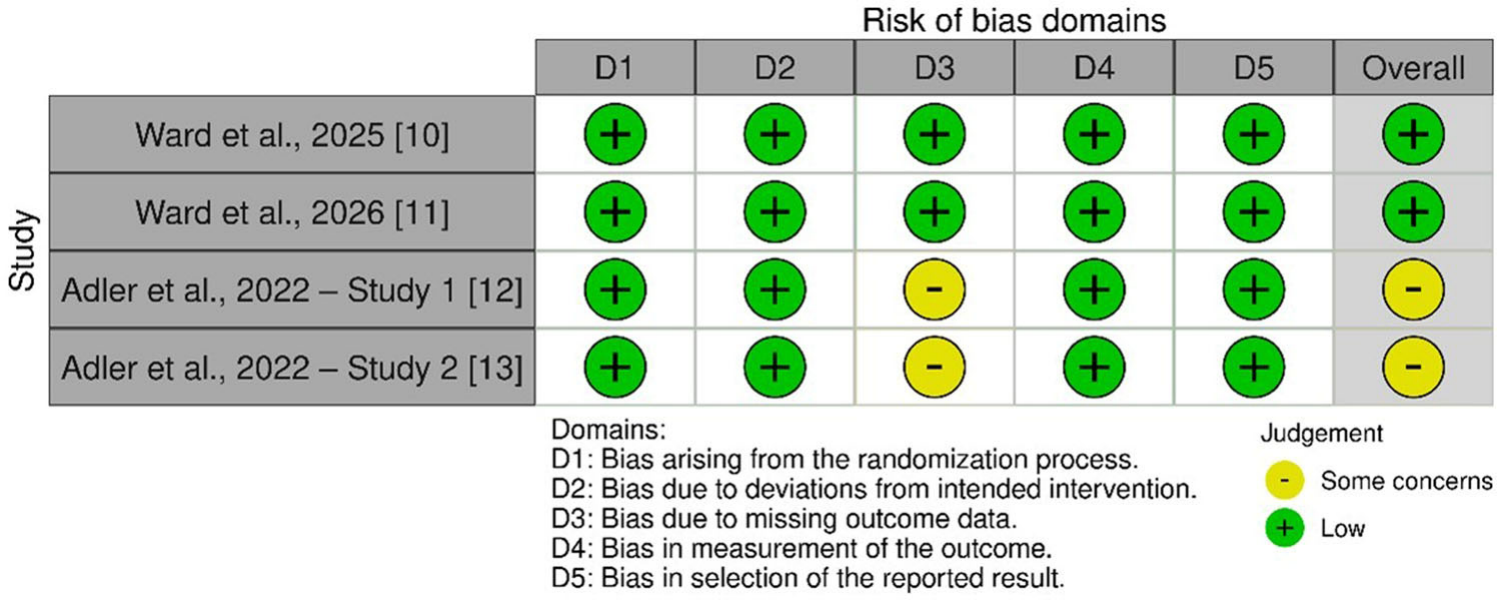
The traffic‐light plot presents the risk‐of‐bias judgments for each included trial across five domains: D1, bias arising from the randomization process; D2, bias due to deviations from intended interventions; D3, bias due to missing outcome data; D4, bias in measurement of the outcome; and D5, bias in selection of the reported result. Green (+) indicates low risk of bias, whereas yellow (−) indicates some concerns.

**Figure 3:**
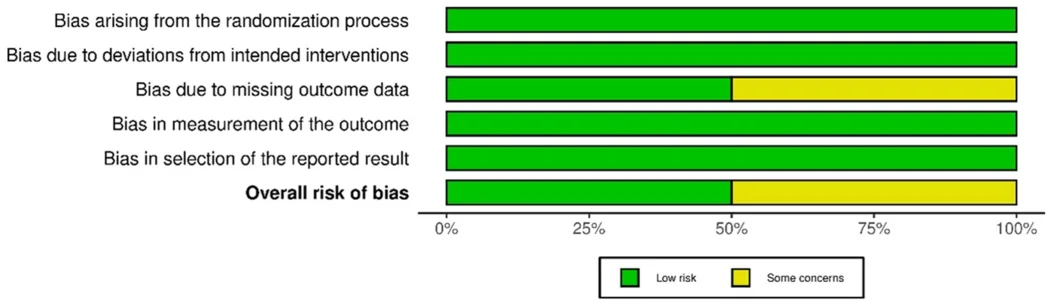
The summary plot presents the proportion of included trials judged as having low risk of bias or some concerns across each Risk of Bias 2 (RoB 2) domain and for the overall risk of bias. Green represents low risk of bias and yellow represents some concerns. No domain was judged to have a high risk of bias.

### Systematic review of outcomes

Three publications reporting four randomized, double‐blind, placebo‐controlled trials were included in the comparative synthesis. Centanafadine was assessed in pediatric, adolescent, and adult populations in the included trials. Adler et al. (2022)^14^ reported two independent Phase 3 trials in adults, named Study 1 (NCT03605680) and Study 2 (NCT03605836), while Ward et al. (2025)^12^ assessed weight‐based low‐ and high‐dose centanafadine in children aged 6–12 years, and Ward et al. (2026)^13^ assessed 164.4‐mg doses in adolescents aged 13–17 years. Due to the lack of a placebo or active comparator, the previously mentioned 52‐week open‐label research was not included in the comparative efficacy analysis. While the adult trials evaluated the primary objective at Day 42, the pediatric and adolescent trials evaluated efficacy after 6 weeks of treatment. Summarized in Table 3.

**Table 3:** Efficacy and safety outcomes of included centanafadine trials.

Outcome	Study	Population/dose	Centanafadine result (LS mean change ± SE/%)	Placebo result (LS mean change ± SE/%)	Statistical findings (effect estimates and *p*‐values)
Change in ADHD symptom severity (ADHD‐RS‐5)	Ward et al., 2025	Children; low‐dose CTN	−13.5 ± 1.21	−10.8 ± 1.21	MD −2.7; 95% CI: −6.0 to 0.5; *p* = 0.10
	Ward et al., 2025	Children; high‐dose CTN	−16.3 ± 1.21	−10.8 ± 1.21	*p* < 0.001
Change in ADHD symptom severity (ADHD‐RS‐5)	Ward et al., 2026	Adolescents; 164.4 mg	−15.5 ± 0.923	−14.2 ± 0.933	*p* = 0.3016; Cohen's *d* = −0.12
	Ward et al., 2026	Adolescents; 328.8 mg	−18.5 ± 0.933	−14.2 ± 0.933	*p* = 0.0006; Cohen's *d* = −0.40
Change in ADHD symptom severity (AISRS)	Adler et al., 2022 (Study 1)	Adults; 200 mg/day	−10.13 ± 0.99 (−25.5%)	−6.98 ± 0.98 (−17.7%)	LSMD −3.15; 95% CI: −5.79 to −0.51; *p* = 0.019; *d* = −0.28
	Adler et al., 2022 (Study 1)	Adults; 400 mg/day	−9.73 ± 0.98 (−24.6%)	−6.98 ± 0.98 (−17.7%)	LSMD −2.74; 95% CI: −5.35 to −0.14; *p* = 0.039; *d* = −0.24
Change in ADHD symptom severity (AISRS)	Adler et al., 2022 (Study 2)	Adults; 200 mg/day	−12.08 ± 0.96 (−32.2%)	−8.07 ± 0.94 (−21.4%)	LSMD −4.01; 95% CI: −6.55 to −1.46; *p* = 0.002; *d* = −0.37
	Adler et al., 2022 (Study 2)	Adults; 400 mg/day	−12.49 ± 0.99 (−32.2%)	−8.07 ± 0.94 (−21.4%)	LSMD −4.42; *p* < 0.001; *d* = −0.40
Change in CGI‐S‐ADHD	Ward et al., 2025	Children; low‐dose CTN	−1.1 ± 0.12	−0.9 ± 0.12	*p* = 0.14
	Ward et al., 2025	Children; high‐dose CTN	−1.1 ± 0.12	−0.9 ± 0.12	*p* = 0.07
Change in CGI‐S‐ADHD	Ward et al., 2026	Adolescents; 164.4 mg	−1.15 ± 0.097	−1.06 ± 0.097	Not significant
	Ward et al., 2026	Adolescents; 328.8 mg	−1.42 ± 0.097	−1.06 ± 0.097	*p* = 0.0038
Change in CGI‐S	Adler et al., 2022 (Study 1)	Adults; 200 mg/day	−0.78 ± 0.096	−0.52 ± 0.086	LSMD −0.27; 95% CI: −0.50 to −0.04; *p* = 0.023
	Adler et al., 2022 (Study 1)	Adults; 400 mg/day	−0.79 ± 0.086	−0.52 ± 0.086	LSMD −0.28; 95% CI: −0.51 to −0.05; *p* = 0.016
Change in CGI‐S	Adler et al., 2022 (Study 2)	Adults; 200 mg/day	−1.04 ± 0.096	−0.71 ± 0.096	LSMD −0.33; 95% CI: −0.57 to −0.09; *p* = 0.007
	Adler et al., 2022 (Study 2)	Adults; 400 mg/day	−0.99 ± 0.096	−0.71 ± 0.096	LSMD −0.28; 95% CI: −0.53 to −0.04; *p* = 0.025
Clinically meaningful response (≥18‐point reduction)	Ward et al., 2025	Children; low‐dose CTN	34%	23%	*p* < 0.05
	Ward et al., 2025	Children; high‐dose CTN	34%	23%	*p* < 0.05
Clinically meaningful response (≥18‐point reduction)	Ward et al., 2026	Adolescents; 164.4 mg	36.0%	31.7%	*p* = 0.4570
	Ward et al., 2026	Adolescents; 328.8 mg	47.7%	31.7%	*p* = 0.0039
ADHD domain: inattention (Conners 3)	Ward et al., 2025	Children; high‐dose CTN	−11.9 ± 0.82	−6.4 ± 0.82	MD −5.5; 95% CI: −8.4 to −2.6; *p* < 0.001
ADHD domain: hyperactivity/impulsivity (Conners 3)	Ward et al., 2025	Children; high‐dose CTN	−11.5 ± 0.92	−6.9 ± 0.92	MD −4.6; 95% CI: −7.5 to −1.7; *p* = 0.002
ADHD domain: executive function (Conners 3)	Ward et al., 2025	Children; high‐dose CTN	−10.4 ± 0.81	−5.8 ± 0.81	MD −4.6; 95% CI: −7.5 to −1.6; *p* < 0.001
ADHD domain: inattention (Conners 3)	Ward et al., 2026	Adolescents; 328.8 mg	−12.84 ± 0.79	−6.21 ± 0.79	MD −6.63; 95% CI: −9.06 to −3.60; *p* < 0.001
ADHD domain: hyperactivity/impulsivity (Conners 3)	Ward et al., 2026	Adolescents; 328.8 mg	−12.03 ± 0.81	−6.51 ± 0.81	MD −5.52; 95% CI: −8.44 to −2.59; *p* < 0.001
ADHD domain: executive function (Conners 3)	Ward et al., 2026	Adolescents; 328.8 mg	−11.47 ± 0.80	−6.55 ± 0.80	MD −4.92; 95% CI: −7.56 to −2.28; *p* < 0.001

Abbreviations: ADHD, attention‐deficit/hyperactivity disorder; ADHD‐RS‐5, attention‐deficit/hyperactivity disorder rating scale, 5th edition; AISRS, Adult ADHD Investigator Symptom Rating Scale; CGI‐S, Clinical Global Impression‐Severity; CGI‐S‐ADHD, Clinical Global Impression‐Severity for ADHD; CI, confidence interval; CTN, centanafadine; *d*, Cohen's *d* effect size; LSMD, least‐squares mean difference; MD, mean difference; SE, standard error.

### Efficacy outcomes

#### Change in ADHD symptom severity

Three studies^12^, ^13^, ^14^ evaluated change in ADHD symptom severity; the primary efficacy outcome was change from baseline in ADHD‐RS‐5 total score in children and adolescents and AISRS total score in adults. Ward et al. (2025)^12^ found that at Week 6, the average ADHD‐RS‐5 total score change was −13.5 ± 1.2 with low‐dose centanafadine and −16.3 ± 1.2 with high‐dose centanafadine, compared to −10.8 ± 1.2 with placebo. Low‐dose centanafadine did not substantially vary from placebo (MD −2.7, 95% CI −6.0 to 0.5; *p* = 0.10). However, high‐dose centanafadine significantly reduced ADHD‐RS‐5 ratings compared to placebo (*p* < 0.001). Ward et al. (2026)^13^ found at Week 6, the ADHD‐RS‐5 total score fell by −15.5 ± 0.92 with centanafadine (164.4 mg) and −18.50 ± 0.93 with 328.8 mg, compared to −14.15 ± 0.93 with a placebo. The 164.4‐mg dose did not differ substantially from placebo (*p* = 0.3016; Cohen's *d* = −0.12), whereas the 328.8‐mg dose resulted in a significant improvement (*p* = 0.0006; Cohen's *d* = −0.40). Adler et al. (2022)^14^ in its Study 1 reported that at Day 42, both doses significantly improved AISRS overall scores compared to placebo. The LS mean difference was −3.15 (95% CI −5.79 to −0.51; *p* = 0.019; Cohen's *d* = −0.28) with 200 mg/day and −2.74 (95% CI −5.35 to −0.14; *p* = 0.039; Cohen's *d* = −0.24) with 400 mg/day. The percentage reduction in AISRS score was 25.5% with 200 mg/day and 24.6% with 400 mg/day, against 17.7% with placebo. Adler et al. (2022) in Study 2^14^ found that both doses performed considerably better than placebo. The LS mean difference was ‐4.01 (95% CI −6.55 to −1.46, *p* = 0.002; Cohen's *d* = −0.37) with 200 mg/day and −4.42 (95% CI −7.02 to −1.82; *p* < 0.001; Cohen's *d* = −0.40) with 400 mg/day. AISRS reductions were 32.2% with both centanafadine doses compared with 21.4% with placebo. Overall, the primary symptom result showed the most evident dose‐related efficacy in children and adolescents, although both 200‐ and 400‐mg/day doses were effective in adults, with no consistent additional efficacy advantage from the higher dose.

#### Change in Clinical Global Impression‐Severity

All three studies^12^, ^13^, ^14^ found the Change in Clinical Global Impression‐Severity. Ward et al. (2025)^12^ found that both low‐ and high‐dose centanafadine reduced CGI‐S‐ADHD scores by −1.1 ± 0.1, compared to −0.9 ± 0.1 for placebo. Neither comparison was statistically significant (low dose, *p* = 0.14; high dose, *p* = 0.07). Ward et al. (2026)^13^ found that CGI‐S‐ADHD scores fell by −1.15 ± 0.09 with 164.4 mg and −1.42 ± 0.09 with 328.8 mg, respectively, compared to −1.06 ± 0.09 with placebo. The 328.8‐mg dose significantly improved CGI‐S‐ADHD compared to placebo (*p* = 0.0038), whereas the 164.4‐mg dose had no statistical significance. Adler et al. (2022) in Study 1^14^ showed the LS mean difference between CGI‐S and placebo was −0.27 (95% CI −0.50 to −0.04; *p* = 0.023) at 200 mg/day and −0.28 (95% CI −0.51 to −0.05; *p* = 0.016) at 400 mg/day. Adler et al. (2022) in Study 2^14^ evaluated CGI‐S also improved significantly with both doses, with an LS mean difference of −0.33 (95% CI −0.57 to −0.09; *p* = 0.007) with 200 mg/day and −0.28 (95% CI −0.53 to −0.04; *p* = 0.025) with 400 mg/day.

#### Clinically meaningful ADHD symptom response

Three studies^12^, ^13^, ^14^ found clinically meaningful ADHD symptom response. Ward et al. (2025)^12^ found that 34% of participants getting low‐dose centanafadine and 34% receiving high‐dose centanafadine saw a ≥18‐point drop in ADHD‐RS‐5 total score, compared to 23% receiving placebo. Both comparisons were statistically significant (*p* < 0.05). The study found that high‐dose centanafadine significantly improved ADHD‐RS‐5 scores by ≥30%, 40%, and 50% compared to placebo (*p* < 0.05). Ward et al. (2026)^13^ found a ≥18‐point reduction in 36.0% with 164.4 mg, 47.7% with 328.8 mg, and 31.7% with placebo. The 164.4‐mg dose was not substantially different from the placebo (*p* = 0.4570), whereas the 328.8‐mg dose was significantly better (*p* = 0.0039). The 328.8‐mg dose significantly enhanced ADHD‐RS‐5 and CGI‐C‐ADHD responses by ≥30%, ≥40%, and ≥50%, respectively (*p* < 0.01). Adler et al. (2022)^14^ defined a responder in adult trials as having a CGI‐C score of 1 or 2 or achieving a 30% improvement in AISRS.

#### ADHD symptom‐domain outcomes

Two studies^12^, ^13^ ADHD symptom‐domain outcomes. Ward et al. (2025)^12^ found that high‐dose centanafadine significantly improved inattention (MD −5.5, 95% CI −8.4 to −2.6; *p* < 0.001), hyperactivity/impulsivity (MD −4.6, 95% CI −7.5 to −1.7; *p* = 0.002), and executive functioning (MD −4.6, 95% CI −7.5 to −1.6; *p* = 0.003) when compared to placebo. Ward et al. (2026)^13^ found that 328.8 mg improved inattention (MD −6.63, 95% CI −9.06 to −3.60; *p* < 0.0001), hyperactivity/impulsivity (MD −5.52, 95% CI −8.44 to −2.59; *p* = 0.0002), and executive functioning (MD −4.92, 95% CI −7.56 to −2.28; *p* = 0.0003).

### Safety outcomes

#### Treatment‐emergent adverse events

Three studies^12^, ^13^, ^14^ evaluating four short‐term trials assessed safety and tolerability. In children, Ward et al. (2025) reported that TEAEs occurred in 52/147 (35%) participants receiving weight‐based low‐dose centanafadine, 61/157 (39%) receiving high‐dose centanafadine, and 39/153 (25%) receiving placebo. Severe TEAEs occurred in 1 (1%) participant in the low‐dose group, 0 in the high‐dose group, and 0 in the placebo group. Discontinuation due to adverse events occurred in 4 (3%), 10 (6%), and 2 (1%) participants, respectively, with no deaths reported. In adolescents, Ward et al. (2026) reported TEAEs in 48/153 (31.4%) participants receiving 164.4 mg, 76/151 (50.3%) receiving 328.8 mg, and 35/147 (23.8%) receiving placebo. Severe TEAEs occurred in 2 (1.3%) participants in the 164.4‐mg group, none in the 328.8‐mg group, and 1 (0.7%) participant in the placebo group. Rash occurred in 3 (2.0%), 9 (6.0%), and 1 (0.7%) participants, respectively. In adults, Adler et al. (2022) reported pooled safety data from two pivotal 6‐week trials. TEAEs occurred in 74/294 (25.2%) participants receiving 200 mg/day, 93/292 (31.8%) receiving 400 mg/day, and 51/290 (17.6%) receiving placebo. Discontinuation due to TEAEs occurred in 14/294 (4.8%), 18/292 (6.2%), and 4/290 (1.4%) participants, respectively.

### Overall findings

Centanafadine showed consistent efficacy for lowering ADHD symptom severity in three publications and four randomized studies, with the most obvious dose‐related pattern seen in children and adolescents. Higher dosages significantly improved ADHD‐RS‐5 scores, CGI‐S‐ADHD, responder outcomes, and symptom‐domain assessments, but lower doses were less consistently superior to placebo. In adults, both 200 and 400 mg/day were significantly efficacious for AISRS and CGI‐S outcomes in two independent trials, with no consistent extra efficacy benefit for the higher dose. Centanafadine was generally well tolerated, however TEAEs and treatment discontinuations increased with increasing dose, particularly in teenagers.

## DISCUSSION

This systematic review is a synthesis of evidence from three publications containing four Phase 3 randomized, double‐blind, placebo‐controlled trials of centanafadine in children, adolescents and adults with ADHD.^15^, ^16^, ^17^ Overall, centanafadine was effective in reducing core ADHD symptoms, with a more pronounced dose–response relationship in children and adolescents, and adults benefiting from both doses tested, with no clear dose–response relationship. These results were largely confirmed by secondary outcomes such as global clinical severity, treatment response, and symptom domains (inattention, hyperactivity/impulsivity, and executive functioning).^16^, ^17^ However, these findings should be interpreted in the context of the small evidence base and the differences in age groups, formulations, and dosing regimens across trials. The overall safety of centanafadine was acceptable, but higher doses were associated with more TEAEs, especially in adolescents. The most frequent side effects were decreased appetite, nausea, headache, and rash, which exhibited a more distinct dose response in children.^16^, ^17^ In general, these results indicate that centanafadine is a potentially effective nonstimulant medication for ADHD, and that the dosing of the medication should be individualized, especially in younger patients, to maximize its effectiveness and tolerability. Overall, these findings support dose optimization according to efficacy and tolerability, particularly in younger patients.

The present findings are consistent with the expanding body of evidence for the first‐in‐class NDSRI centanafadine. The previous systematic review evidence showed that centanafadine has modest but significant reductions in ADHD symptom severity and global clinical status compared to placebo.^18^ The results are consistent with the efficacy seen in the adult and higher dose pediatric trials used in this review.^15^, ^16^, ^17^ Nevertheless, the relatively small number of available trials limits the certainty with which these findings can be generalized across age groups.

Furthermore, based on comparative evidence from matching‐adjusted indirect comparisons, the tolerability profile of centanafadine may be favorable relative to a number of established ADHD pharmacotherapies, such as lisdexamfetamine, atomoxetine and viloxazine ER.^19^ These comparisons are indirect and were not part of the randomized evidence synthesized in the present review; therefore, they should not be interpreted as demonstrating superior safety or tolerability. These results, however, are indirect comparisons and should be interpreted with caution. Centanafadine might have tolerability benefits for some side effects, but its efficacy compared to stimulant treatments is less clear, and further head‐to‐head studies are needed. The included pediatric and adolescent studies also reported improvements in selected ADHD symptom domains, including inattention, hyperactivity/impulsivity, and executive‐function‐related measures.^16^, ^17^ However, these findings should be interpreted as secondary or domain‐specific efficacy outcomes rather than evidence of broader improvements in executive functioning, emotional regulation, learning, or daily task performance. The present review did not consistently evaluate these broader functional outcomes across all included trials. Therefore, claims regarding broader cognitive or functional benefits should remain cautious until adequately powered studies specifically designed to assess these outcomes are available.

Notably, the dose–response pattern observed in this review is consistent with evidence suggesting that treatment response may vary with dose across ADHD pharmacotherapies.^8^ The dose–response relationship appeared more pronounced in children and adolescents than the relatively similar effects observed across the adult doses evaluated, which may reflect differences in pharmacokinetics, treatment exposure, receptor sensitivity, or ceiling effects.^20^ These differences require further investigation through age‐specific pharmacokinetic and dose‐ranging studies. The included pediatric and adolescent studies also reported changes in selected domains, including executive functioning, inattention, and hyperactivity/impulsivity.^8^, ^16^, ^20^ However, the available evidence is insufficient to determine whether these findings translate into improvements in emotional regulation, learning, or broader daily functioning. Further studies specifically designed to assess these outcomes are warranted.

Moreover, the safety results were similar to those seen in previous Phase 2 and Phase 3 studies.^15^, ^19^, ^20^ The adverse events were more common with active treatment and higher doses of centanafadine, although they were generally well tolerated; rash was more prominent in children Rash was reported more frequently in younger populations and should therefore be considered when monitoring tolerability, particularly during dose escalation. Importantly, the present review cannot establish that centanafadine is safer than existing ADHD therapies because none of the included randomized trials directly compared centanafadine with an active stimulant or nonstimulant comparator. Similarly, potential differences in gastrointestinal, sexual, or other adverse effects reported in indirect comparisons should be regarded as hypothesis‐generating rather than evidence of comparative superiority.^19^, ^20^ Overall, these results indicate that centanafadine could be a valuable addition to the growing list of nonstimulant ADHD medications. Longer term and head‐to‐head studies are needed to establish the comparative safety and tolerability of centanafadine.

However, the results indicate that the dose–response relationships for the different age groups are divergent, which has clinical implications. In children and adolescents, careful dose optimization may be required to ensure that the dose is optimized for symptom control and to reduce TEAEs. However, the similar efficacy observed across the doses evaluated suggests that dose escalation should be guided by individual clinical response and tolerability rather than assuming that higher doses provide greater efficacy. The available evidence does not allow conclusions regarding specific benefits of centanafadine for emotional regulation, learning, or broader functional impairment. The similar efficacy seen across the doses evaluated, and increased tolerability at lower doses, suggest that dose escalation in adults should be done individually. The reported changes in executive function indicate that centanafadine could be especially important for individuals with high levels of cognitive and functional impairments.^8^, ^17^, ^19^, ^20^ Moreover, its dual catecholaminergic and serotonergic mechanism may be beneficial in patients with certain psychiatric comorbidities. These potential benefits, however, have yet to be confirmed in sufficiently powered, prospective, comparative studies. In adults, the similar efficacy observed across the doses evaluated suggests that dose escalation should be guided by individual clinical response and tolerability rather than assuming that higher doses provide greater efficacy. The available evidence does not allow conclusions regarding specific benefits of centanafadine for emotional regulation, learning, or broader functional impairment. Moreover, although its multimodal NDSRI mechanism provides a pharmacologically distinct approach to ADHD treatment, whether this mechanism offers advantages in specific clinical subgroups or in patients with psychiatric comorbidities remains uncertain and requires prospective investigation. Overall, centanafadine was generally well tolerated in the included trials, but the dose‐related increase in TEAEs and discontinuations, particularly in adolescents, emphasizes the importance of individualized dosing and monitoring.^16^, ^17^, ^20^ Because the controlled trials were predominantly short‐term, longer term studies are necessary to establish the durability of efficacy and the longer term safety profile.

### Strengths and limitations

This systematic review synthesized available Phase 3 randomized, double‐blind, placebo‐controlled evidence for centanafadine in children, adolescents, and adults with ADHD and specifically examined dose–response patterns alongside efficacy, safety, and tolerability. The methodological rigor, including application of PRISMA 2020 guidelines and independent study selection, data extraction, and RoB 2 assessment, enhances the credibility of the findings. Multiple age groups and dosing regimens allowed clinically relevant assessment of age‐ and dose‐related treatment patterns.

Only three publications containing four randomized trials were available, which limited comprehensive subgroup analyses and evaluation of uncommon adverse events. There was no quantitative meta‐analysis possible, and direct comparisons between studies were limited by differences in age groups, dosing regimens, outcome measures, and follow‐up periods. The trials were predominantly short‐term, and therefore the results cannot be used to draw firm conclusions regarding long‐term efficacy, safety, or treatment persistence. In addition, there were no active‐comparator trials, limiting assessment of the comparative effectiveness and safety of centanafadine versus established stimulant and nonstimulant ADHD treatments. Importantly, all included randomized trials appear to have been sponsored by the pharmaceutical company developing centanafadine. The absence of independently sponsored randomized evidence represents an additional limitation and introduces the possibility of sponsorship‐related bias. Accordingly, the efficacy and safety findings, as well as any potential advantages over existing ADHD therapies, should be interpreted cautiously until supported by independent research.

### Future implications

Future research should prioritize long‐term studies to establish the durability of centanafadine's efficacy and safety across different age groups. Head‐to‐head trials against established stimulant and nonstimulant ADHD therapies are needed to determine its comparative effectiveness and tolerability. Independent, investigator‐initiated studies are also needed to confirm the efficacy and safety findings observed in the predominantly industry‐sponsored evidence base. Further pharmacokinetic and pharmacogenetic studies may help clarify age‐ and dose‐related differences and optimize individualized dosing. Real‐world studies should also evaluate adherence, quality of life, and functional outcomes, including academic and occupational performance.

## CONCLUSION

The present systematic review indicates that centanafadine improves core ADHD symptoms in children, adolescents, and adults, with a more pronounced dose–response pattern observed in children and adolescents and significant efficacy across the doses evaluated in adults. Higher doses were associated with increased treatment‐emergent adverse‐event rates in some populations. However, the evidence base remains limited to four randomized trials from three publications, with predominantly short‐term follow‐up and no active‐comparator trials. In addition, the included trials appear to have been sponsored by the manufacturer, while independently sponsored evidence remains limited. Therefore, findings regarding efficacy, safety, and dose–response should be interpreted cautiously. The current evidence does not establish superior safety compared with other ADHD treatments or demonstrate specific efficacy for emotional regulation, learning, or broader functional outcomes. Longer term, independently sponsored, and head‐to‐head comparative trials are needed to further establish the optimal dosing, comparative effectiveness, and long‐term safety profile of centanafadine.

## AUTHOR CONTRIBUTIONS

**Daud Gul**: Conceptualization, study design, and overall supervision of the project. **Sonia Khan**: Data collection and methodological assistance. **Kushal Baral**: Statistical analysis, data validation, and interpretation of results. **Ziad Saber**: Literature review and drafting of the Introduction and Discussion sections. **Arindam Ghosh**: Data acquisition and manuscript organization. **Eemaz Nathaniel**: Contribution to the Discussion and manuscript refinement. **Onuwabhagbe Daniel Omomhenle**: Clinical input and review of psychiatric relevance. **Nathaniel Oluwakayode Oyedeji**: Manuscript editing and review of results interpretation. **Lana Omar Saed Alkhatib**: Review of clinical and pharmacological implications and proofreading. **Lina Jamal Amin Sa'doun**: Literature review support and referencing. **Austin Nwawueze**: Drafting of the Methods section and contribution to the Discussion. **Muhammad Naveed Ullah Khan**: Critical review and manuscript refinement. **Raouf Khan**: Contribution to data interpretation, critical review of the manuscript, and overall scientific input. All authors meet the ICMJE authorship criteria, approved the final version of the manuscript, and agree to be accountable for all aspects of the work.

## CONFLICT OF INTEREST STATEMENT

The authors declare no conflicts of interest.

## ETHICS APPROVAL STATEMENT

This study was a systematic review of previously published studies and did not involve direct recruitment or participation of human subjects. Therefore, institutional ethical approval was not required.

## PATIENT CONSENT STATEMENT

Informed consent was not required because this study analyzed data from previously published studies and did not involve direct participant recruitment or interaction.

## CLINICAL TRIAL REGISTRATION

This study was a systematic review and was not a clinical trial; therefore, ClinicalTrials.gov registration is not applicable. The review protocol was prospectively registered with PROSPERO (CRD420261475007).

## Supporting information

Supplementary Information

## Data Availability

The data supporting the findings of this study are available from the corresponding author upon reasonable request.
