# Supplementary material for: Dose‐dependent efficacy and safety of centanafadine in patients with attention‐deficit/hyperactivity disorder: A systematic review of the latest evidence
Source: PCN Rep. 2026 Oct 14;5(4):e70428. doi: 10.1002/pcn5.70428 (PMC13664669; doi:10.1002/pcn5.70428)
Supplement: Supplementary file 1 [file PCN5-5-e70428-s001.docx]

**SUPPLEMENTARY FILE**

**PUBMED=29**

("centanafadine"[All Fields] OR "EB-1020"[All Fields])AND("Attention Deficit Disorder with Hyperactivity"[MeSH Terms]OR "ADHD"[All Fields]OR "attention deficit hyperactivity disorder"[All Fields]OR "attention-deficit hyperactivity disorder"[All Fields]OR "attention deficit disorder with hyperactivity"[All Fields]

**Embase =82**

(centanafadine:ti,ab,kw OR "centanafadine hydrochloride":ti,ab,kw OR "centanafadine XR":ti,ab,kw OR "EB-1020":ti,ab,kw OR "DOV 216,419":ti,ab,kw AND (ADHD:ti,ab,kw OR "attention deficit hyperactivity disorder":ti,ab,kw OR "attention-deficit hyperactivity disorder":ti,ab,kw OR "attention deficit disorder with hyperactivity":ti,ab,kw)

**Scopus=29**

( TITLE-ABS-KEY ( centanafadine OR "centanafadine hydrochloride" OR "centanafadine XR" OR "EB-1020" OR "DOV 216,419" ) AND TITLE-ABS-KEY ( ADHD OR "attention deficit hyperactivity disorder" OR "attention-deficit hyperactivity disorder" OR "attention deficit disorder with hyperactivity" ) )

**Cochrane =14**

| **Search** | **Search terms** |
| --- | --- |
| **#1** | centanafadine OR "centanafadine hydrochloride" OR "centanafadine XR" OR "EB-1020" OR "DOV 216,419" |
| **#2** | ADHD OR "attention deficit hyperactivity disorder" OR "attention-deficit hyperactivity disorder" OR "attention deficit disorder with hyperactivity" |
| **#3** | #1 AND #2 |
